# CS-PHOC: weekly census counts of Southern Ocean phocids at Cape Shirreff, Livingston Island

**DOI:** 10.1038/s41597-024-03744-9

**Published:** 2024-08-17

**Authors:** Samuel M. Woodman, Renato Borras-Chavez, Michael E. Goebel, Daniel Torres, Anelio Aguayo, Douglas J. Krause

**Affiliations:** 1 U.S. Antarctic Marine Living Resources Program, Ecosystem Science Division, Southwest Fisheries Science Center, NOAA Fisheries, La Jolla, California, USA; 2 Department of Biology, Baylor University, Waco, Texas USA; 3 Scientific Department, Chilean Antarctic Institute (INACH), Punta Arenas, Chile; 4 Antarctic Ecosystem Research Division, Southwest Fisheries Science Center, NOAA Fisheries, La Jolla, California, USA; 5 Institute of Marine Sciences, University of California Santa Cruz, Santa Cruz, California USA

**Keywords:** Population dynamics, Conservation biology

## Abstract

Rapid climatic warming of the Antarctic Peninsula is driving regional population declines and distribution shifts of predators and prey. Affected species include Antarctic ice seals and the southern elephant seal, all of which rely on the peninsula region for critical stages of their life cycle. However, data collection is difficult in this remote region, and therefore long-term time series with which to identify and investigate population trends in these species are rare. We present the Cape Shirreff Phocid Census (CS-PHOC) dataset: weekly counts of phocids (crabeater, leopard, southern elephant, and Weddell seals) hauled out at Cape Shirreff, Livingston Island, during most austral summers since 1997. Data from these censuses were cleaned and aggregated, resulting in robust and comparable count data from 284 censuses across 23 field seasons. The CS-PHOC dataset, which is publicly available through the SCAR Biodiversity Portal, will be updated yearly to provide important information about Southern Ocean phocids in the Antarctic Peninsula.

## Background & Summary

The Antarctic Peninsula (AP) is one of the most rapidly warming regions in the world^1,2^. Increases in air and sea temperatures in recent decades along the western and northern AP have reduced sea ice extent both spatially and temporally^3,4^. Warming sea water together with the loss of sea ice are expected to shift the regional distributions of pelagic communities, including Antarctic krill (*Euphausia superba*, hereafter krill), myctophids, Antarctic silverfish (*Pleuragramma antarctica*, hereafter silverfish), and their myriad dependent vertebrate predators^5–8^. Whether there is a trend in the total biomass of krill within the AP is debated^9–13^; however, evidence suggests that the krill population is contracting southward and away from traditional krill predator foraging hot spots in the northern AP^14^. Indeed, over the last 20 years, krill and myctophids have become less available to some regional predators^15,16^. Further, a growing krill fishery that is temporally and spatially concentrating its effort^17^, along with regionally recovering baleen whale populations^18,19^, may be decreasing local prey availability and exacerbating various climate effects. Collectively these factors may help explain the substantial decreases in the abundance of regional krill- and fish-dependent species, including ice-associated penguins^20,21^ and Antarctic fur seals (*Arctocephalus gazella*)^15^.

Antarctic ice seals, including crabeater (*Lobodon carcinophaga*), leopard (*Hydrurga leptonyx*), and Weddell (*Leptonychotes weddellii*) seals, as well as the southern elephant seal (*Mirounga leonina*), are important components of Southern Ocean ecosystems as apex predators and major consumers of the above-listed pelagic forage species. The AP in particular is an essential habitat for ice seals, where the density of these predators is higher than other surveyed areas of the continent^22^. Crabeater seals are extremely numerous, and as krill specialists may be the largest marine mammal consumer of krill in the AP^23,24^. Although in East Antarctica southern elephant seal diet is mostly composed of cephalopods, in the northern AP their diet consists primarily of myctophids^25,26^. Leopard and Weddell seals depend on krill, myctophids, and silverfish to varying degrees based on region, sex, mass, and time of year^27–30^. Given the broad-scale changes in ice habitat, temperatures, and the availability of prey in the AP, extensive changes in the population dynamics and distribution of these southern phocids are both predicted^23,31,32^ and have been observed^15^. However, a suite of unique challenges, including remote pack-ice environments and periodic haul-outs, makes AP phocids difficult to survey^23,33,34^. Therefore, changes in their population dynamics are extremely difficult to detect using the few existing population counts, which have large associated uncertainties^22^.

In the northern AP, Cape Shirreff, Livingston Island, is an important breeding and resting site for Southern Ocean phocids and other krill predators^15,35^. As such, it has been recognized by the Antarctic Consultative Treaty Meeting as an Antarctic Specially Protected Area^36^. As part of long-term monitoring efforts at Cape Shirreff, the National Oceanic and Atmospheric Administration Fisheries (NOAA Fisheries) United States Antarctic Marine Living Resources Program (U.S. AMLR Program) and the Chilean Antarctic Institute (INACH) have conducted synoptic, weekly counts of Southern Ocean phocids hauled out on Cape Shirreff during most austral summers since 1997/98. Here, we present these census data, which will continue to be collected by the U.S. AMLR Program and thus updated yearly. This dataset provides a rare and valuable source of information about changes in population trends and area use by Southern Ocean phocids in a climate change hot spot.

## Methods

### Survey methods

**Fig. 1:**
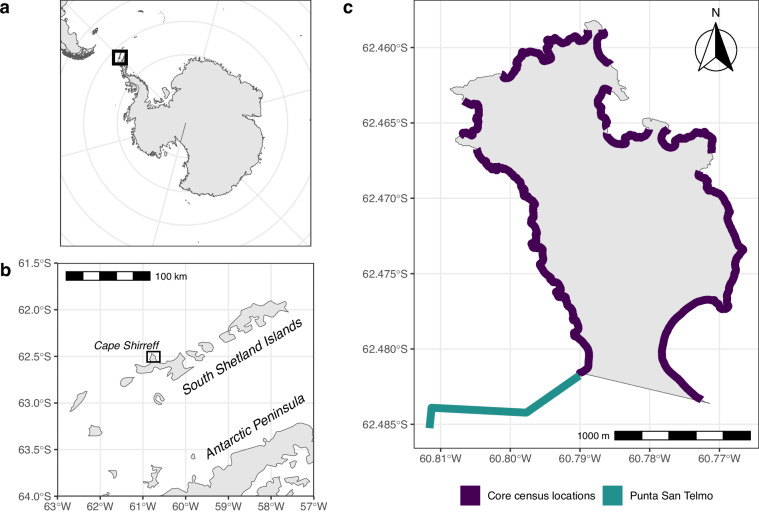
Maps of the study site and the surrounding region: the South Shetland Islands and Cape Shirreff (**a,b**), and regular CS-PHOC survey locations as thick lines along the coast, shaded dark purple for the core census locations and aquamarine for Punta San Telmo (**c**).

**Fig. 2:**
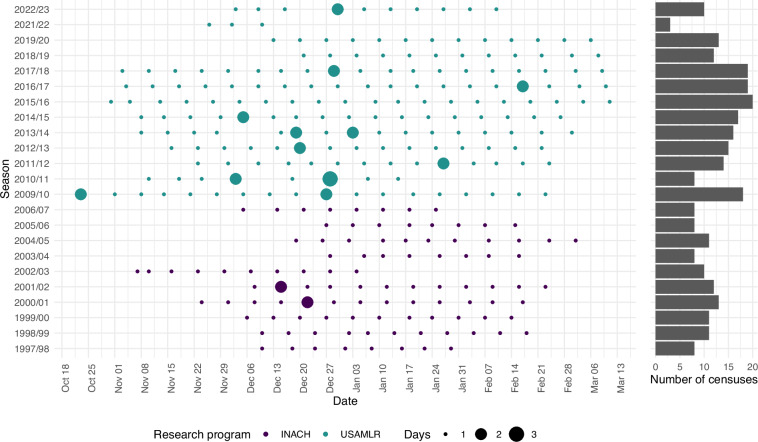
Summary of all CS-PHOC survey windows. Shown are survey window start dates and lengths, as well as the research program that conducted the survey. The right graph shows the number of surveys (i.e., censuses) performed in each season. There were no surveys in 2007/08 and 2008/09 due to program transition, and no field season in 2020/21 due to the COVID-19 pandemic.

All data were collected at Cape Shirreff (62.47° S, 60.77°W) on the north shore of Livingston Island (Fig. 1). Bounded by glaciers to the south, Cape Shirreff is approximately 3 km long and 1.5 km wide. The Cape Shirreff Phocid Census (CS-PHOC, pronounced ‘Seasfolk’) surveys were conducted by INACH from 1997/98 to 2006/07. The U.S. AMLR Program resumed these surveys in 2009/10, and, except for 2020/21 when the field season was cancelled due to the COVID-19 pandemic, have continued surveys every season since through the time of publication. Most CS-PHOC survey windows (i.e., censuses) were only one day, meaning surveys of all locations were conducted on the same day. However, censuses occasionally spanned two or three days due to extenuating circumstances (e.g., weather; Fig. 2).

The INACH and U.S. AMLR programs both followed the same overall census protocol, where trained field biologists surveyed safely accessible regions of Cape Shirreff and recorded all live phocids. Biologists used field notebooks to record counts of each phocid species at each location, along with age class and sex when possible. Phocids were only recorded if they were hauled out on land, and not if they were for instance on an ice flow or swimming just offshore. After the survey, data were entered into a database or otherwise archived. Locations were surveyed on foot, by either walking through haul-out locations or using binoculars from a high vantage point where practical. While the full extent of the area surveyed varied slightly both across and within seasons, core census locations were always surveyed. These core census locations span the vast majority of phocid haul-out locations at Cape Shirreff (Fig. 1), thereby ensuring that CS-PHOC data are both consistent and representative of phocid haul-out at Cape Shirreff across all censuses and seasons.

### Data cleaning and aggregation

Data records were compiled from historical documents, field notebooks, Excel files, and a SQL Server database. INACH historical data (i.e., paper records, reports, and Excel files) were consolidated into Excel files. These INACH files, along with historical U.S. AMLR Excel files, were imported into the U.S. AMLR Pinniped SQL Server database using R (v4.3)^37^. Once in the database, all data were read into R, where they were cleaned and standardized as follows. Location names and count types (i.e., age class and sex) were converted to standard names, and columns containing count data were aggregated to the lowest resolution across datasets. For instance, some seasons male and female pup counts were recorded separately, but during others only a single count of all pups was recorded. Thus, for this dataset, all pup counts were aggregated to a single, total pup count for each survey record. In addition, recorded data varied slightly across programs. Specifically, data from INACH surveys included explicit zero records when there were none of a particular phocid species at a location, while data from U.S. AMLR surveys only included non-zero counts. To make these data consistent, U.S. AMLR data were made explicit by adding zero records as necessary.

After data cleaning and standardization, records from the core census locations were grouped and aggregated to provide a single, comparable count for each species for each census. Specifically, records were filtered for core census locations, and counts were summed after grouping by census and species. These aggregated core census location counts, along with counts for one other location (Punta San Telmo, described in “Data Records” below; Fig. 1), make up the published CS-PHOC dataset.

### Data publication

The CS-PHOC dataset described in this manuscript has been made Darwin Core compliant, and published as open data accessible via the SCAR Antarctic Biodiversity Portal (www.biodiversity.aq). This ensures that the data is broadly available through the Ocean Biodiversity Information System (OBIS) and the Global Biodiversity Information Facility (GBIF). Data from future field seasons will be added once it has been cleaned and processed, ensuring that the published CS-PHOC dataset remains up to date for future analyses.

## Data Records

The CS-PHOC dataset is available as a Darwin Core (DwC) Archive via www.biodiversity.aq^38^. These data were generated from two CSV files, cs-phoc-events.csv and cs-phoc-counts.csv, using R scripts included in the CS-PHOC GitHub repository (https://github.com/us-amlr/cs-phoc; 10.5281/zenodo.12735249)^39^. Again, the published dataset is explicit, meaning that records with a numeric value exist if and only if field biologists conducted a count for that specific location, species, and count type. If such a record does not exist for a census, then either that location was not surveyed or that particular count type was not recorded for that species at that time. The DwC Archive dataset consists of event and occurrence (i.e., count) records, which can be joined using the eventID values present in both sets of records. While these records use standard DwC terms (https://dwc.tdwg.org/terms/), users should note the following for the event records: the eventRemarks field describes if a survey spanned multiple days, the research program that conducted the surveys (i.e., ‘INACH’ or ‘USAMLR’) is indicated using the dynamicProperties field, and the location (i.e., core census locations or Punta San Telmo) of an event is captured in both the locality and locationRemarks fields.

Field biologists generally split out the core census locations into smaller areas for surveys; however, the exact boundaries between those areas sometimes varied slightly across field seasons. As described in the Methods section, the core census locations consist of all the locations on Cape Shirreff that were surveyed consistently by both the INACH and U.S. AMLR programs. Thus, the dataset described in this manuscript consists of only the counts comparable across seasons: 1) aggregated counts for all core census locations and 2) counts for the Punta San Telmo location. The core census location counts span the entire timeseries, while the counts for Punta San Telmo are also included in this dataset because this location has been included in most surveys since the 2009/10 field season (n = 177 out of 184 surveys).

## Technical Validation

All event records were reviewed and confirmed using field notebook scans. All count records were screened for unreasonable values or duplicate entries via R code, either programmatically or visually through plots of the data. Duplicates were removed, and other data flagged by automated checks were validated using paper datasheets or scans of field notebooks. Count records were also checked for consistency with regard to zero vs NA values, ensuring that patterns in the data were consistent (e.g., values for a particular count type were either all NA or all non-NA for a full season). All observed patterns, as well as consistent survey scope and techniques over the full timeseries, were confirmed by research directors.

## Usage Notes

The authors advise users of these data to be aware that there are likely many intrinsic and extrinsic drivers of phocid haul-out behaviour at Cape Shirreff, other than simply regional abundance of a particular species. For example, regional census counts are greatly influenced by life history traits such as the timing of breeding and moulting, or congregations around prey populations. Southern elephant seals have well-established haul out patterns to breed and moult, and these patterns vary across sex, age class, and breeding state^40,41^. Like southern elephant seals, Weddell seals also regularly pup at or near Cape Shirreff between late September and early December (U.S. AMLR, unpublished data) and exhibit seasonal haul-out patterns^42^. In addition, seasonally resident leopard seals frequent Cape Shirreff from late December through February, likely to prey on locally-breeding penguins and Antarctic fur seals^43^. These patterns are reflected in the CS-PHOC counts, and must be taken into consideration when drawing conclusions from these data.

**Fig. 3:**
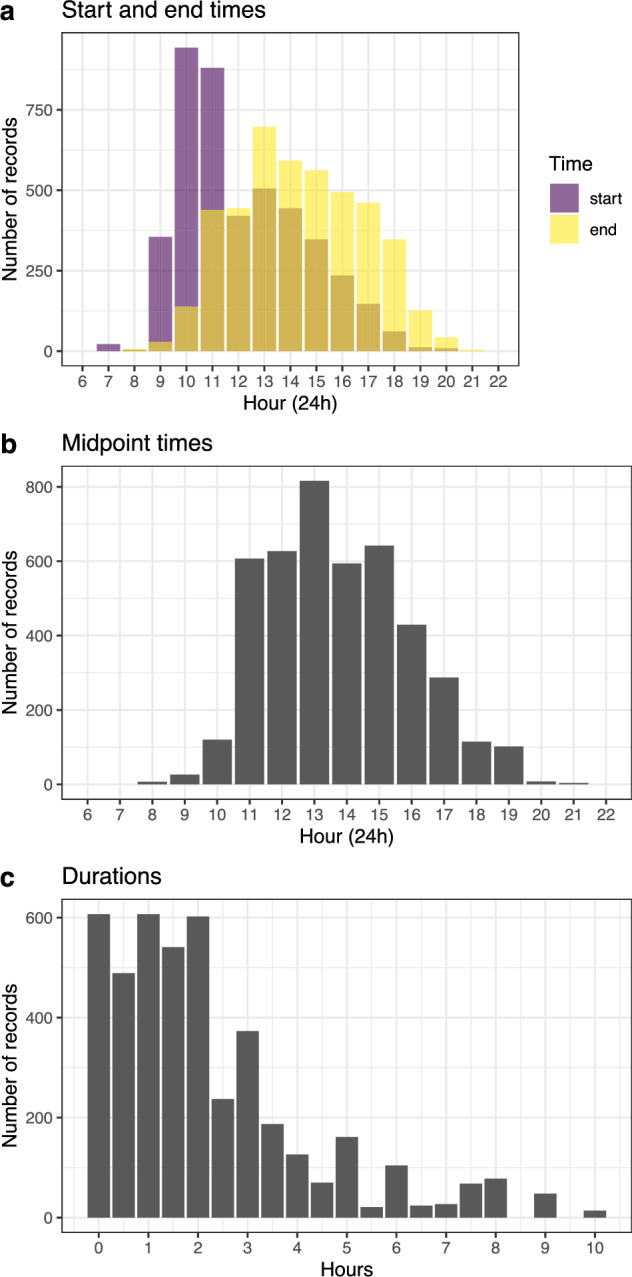
Overview of available time of day information from all single-day CS-PHOC survey records with start and end times (n = 4066 records). Times, which were recorded for most surveys starting in 2009, were sometimes recorded for individual locations (less than one hour), and sometimes for some or all of a survey effort (up to ten hours). (**a**) Survey record start and end times, by hour. (**b**) The midpoint time, rounded to the nearest hour, of all survey records with start and end times. (**c**) The length of time (i.e., duration) of survey records with start and end times.

Other factors can also influence phocid haul-out probabilities, including meteorological conditions (especially those that affect air temperature), tides, or time of day^22,42–44^. These factors must be carefully considered when using these data. Methods exist to correct for these factors in regional census data (e.g.^22^); however, the presented CS-PHOC count records do not have the necessary precise date or time information. This is due to the aggregation of data from multi-day surveys, and because survey times were historically recorded inconsistently (i.e., sometimes for a single beach, and sometimes for a full day’s survey effort over multiple locations). While implementing haul-out corrections is thereby currently impractical, CS-PHOC surveys were typically conducted in the middle of the day (between 1000 and 1500 local time) to maximize sighting probabilities for all species (Fig. 3). Survey start and end times have been recorded for each individual location since the start of the 2021/22 season, and implementing time-based haul-out corrections will be possible with future data. In summary, the CS-PHOC data should be interpreted cautiously because of the short-term variability in many haul-out drivers; however, we are confident that the long-term counts presented in this dataset can be used to make useful inference, given both the relative consistency of survey times and the breadth of the timeseries.

Since the CS-PHOC survey windows sometimes spanned multiple days, we recommend using the census start date as the date for each count record. This is reflected in the DwC Archive dataset, where the eventDate field is the census start date for all multiday censuses.

**Fig. 4:**
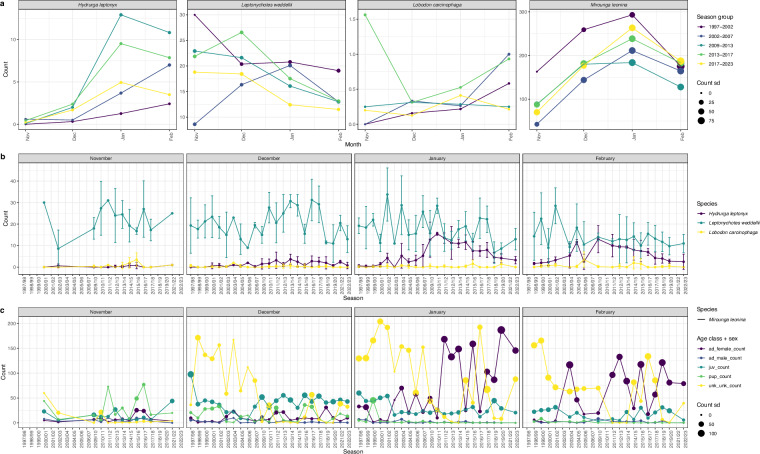
Example visualizations of CS-PHOC data. (**a**) Mean counts for all species, averaged by month and season group, and with standard deviation (sd) represented by dot size. (**b**) Mean counts for leopard, Weddell, and crabeater seals, averaged by month for each season, with error bars showing the standard deviation. Southern elephant seals are presented separately because of their much higher count values. (**c**) Mean counts for southern elephant seals by sex and age class, averaged by month for each season, and with standard deviation represented by dot size.

As described in the Data Records section, the only counts that can be compared across the full timeseries (i.e., since 1997/98) are the counts for the core census locations. Examples of possible ways to visualize and explore these data for the full timeseries are shown in Fig. 4. Analyses including counts for Punta San Telmo should only include data from the 2009/10 field season and later; example code for filtering for this time period and aggregating counts is provided in the GitHub repository. Parties with general questions about these data, or those interested in exploring either finer resolution survey data or available start and end time information, may contact the corresponding author. Users should also note that this data descriptor manuscript was peer reviewed in 2024 based on the data and code available at the time (https://ipt-obis.gbif.us/resource?r=usamlr_cs-phoc&v=2.0; 10.5281/zenodo.12735249)^39^.

## Data Availability

All files and code for importing, cleaning, and processing the CS-PHOC data described in this manuscript, as well as code for generating the figures and example processing, is available at https://github.com/us-amlr/cs-phoc.
